# Life or death by NF*κ*B, Losartan promotes survival in *dy*^*2J*^*/dy*^*2J*^ mouse of MDC1A

**DOI:** 10.1038/cddis.2015.60

**Published:** 2015-03-12

**Authors:** M Elbaz, N Yanay, S Laban, M Rabie, S Mitrani-Rosenbaum, Y Nevo

**Affiliations:** 1 Pediatric Neuromuscular Laboratory and Neuropediatric Unit, Hadassah – Hebrew University Medical Center, Jerusalem, Israel; 2 Goldyne Savad Institute of Gene Therapy, Hadassah – Hebrew University Medical Center, Jerusalem, Israel; 3 Institute of Neurology, Schneider Children's Medical Center of Israel, 14 Kaplan St., Petach Tikva, Israel

## Abstract

Inflammation and fibrosis are well-defined mechanisms involved in the pathogenesis of the incurable Laminin *α*2-deficient congenital muscular dystrophy (MDC1A), while apoptosis mechanism is barely discussed. Our previous study showed treatment with Losartan, an angiotensin II type I receptor antagonist, improved muscle strength and reduced fibrosis through transforming growth factor beta (TGF-*β*) and mitogen-activated protein kinases (MAPK) signaling inhibition in the *dy*^*2J*^*/dy*^*2J*^ mouse model of MDC1A. Here we show for the first time that Losartan treatment up-regulates and shifts the nuclear factor kappa B (NF*κ*B) signaling pathway to favor survival *versus* apoptosis/damage in this animal model. Losartan treatment was associated with significantly increased serum tumor necrosis factor alpha (TNF-*α*) level, p65 nuclei accumulation, and decreased muscle IκB-*β* protein level, indicating NF*κ*B activation. Moreover, NF*κ*B anti-apoptotic target genes TNF receptor-associated factor 1 (*TRAF1*), TNF receptor-associated factor 2 (*TRAF2*), cellular inhibitor of apoptosis (*cIAP2*), and Ferritin heavy chain (*FTH1*) were increased following Losartan treatment. Losartan induced protein expression toward a pro-survival profile as BCL-2 expression levels were increased and Caspase-3 expression levels were decreased. Muscle apoptosis reduction was further confirmed using terminal deoxynucleotidyltransferase-mediated dUTP nick end labeling (TUNEL) assay. Thus, along with TGF-*β* and MAPK signaling, NF*κ*B serves as an important regulatory pathway which following Losartan treatment promotes survival in the *dy*^*2J*^*/dy*^*2J*^ mouse model of MDC1A.

Congenital muscular dystrophy type 1A (MDC1A) is one of the most common forms of congenital muscular dystrophies (CMDs). Clinical symptoms are severe hypotonia, muscle weakness, and delayed motor milestones.^1^ Typically, the children do not achieve independent ambulation and respiratory failure is followed by death in the second or third decade of life.^2, 3^ MDC1A is caused by mutations in the *LAMA2* gene, encoding the heavy chain of laminin-2.^4^ Muscle biopsies are characterized by muscle fiber necrosis, inflammation, apoptosis, and fibrosis.^1, 2, 3, 5^ Despite extensive advances in its diagnosis, MDC1A remains an incurable disease.^2, 6^

The *dy*^*2J*^*/dy*^*2J*^ mouse is a useful model to study the pathophysiology of MDC1A and the effect of various therapeutic agents.^7, 8, 9^ This mouse has a mutation in the *LAMA2* gene resulting in abnormal splicing of the laminin-*α*2 polypeptide and a moderate to severe phenotype characterized by development of muscle weakness at about 3 weeks of age, which progressively worsens.^7, 10^ The pathology of *dy*^*2J*^*/dy*^*2J*^ skeletal muscle is quite similar to children with MDC1A, showing muscle fiber degeneration, necrosis, and apoptosis, followed by inflammation and fibrosis.^7, 10, 11, 12^

Previous studies among ours have shown that the pathogenesis of muscular dystrophies involves coordinated activation of multiple key signaling pathways.^9, 13, 14, 15, 16^ Nuclear factor kappa B (NF*κ*B) has been described as a significant transcription factor that regulates the expression of muscle proinflammatory cytokines.^16^

Early studies have shown elevated NF*κ*B levels in skeletal muscle of *mdx* mice, the mouse model for Duchenne muscular dystrophy (DMD), and in inflammatory myopathies.^17, 18, 19, 20, 21^ NF*κ*B activation is thought to contribute to the deterioration of skeletal muscle pathology and muscle loss in DMD.^18^ However, NFκB seems to have a multifaceted regulatory role and may show protective activity in different disorders. Baghdiguian *et al.*^22, 23^ have shown that a certain level of NF*κ*B activity is required to protect myofibers from apoptosis in a Calpain-3 mouse model of Limb girdle muscular dystrophies (LGMDs). Several studies indicate that NF*κ*B activation has a positive role in cell survival by inducing transcription of several survival genes.^24, 25^ However, there is only limited data regarding NF*κ*B's role in MDC1A.

Losartan, an angiotensin II type 1 receptor antagonist, is a commercially available and extensively used medication for hypertension with a low side effect profile, occasionally used in childhood.^26^ In our previous study, we showed that Losartan treatment was associated with significant impressive improvement in muscle strength and amelioration of fibrosis in the *dy*^*2J*^*/dy*^*2J*^ mouse model of MDC1A, through inhibition of transforming growth factor beta (TGF-*β*) and the mitogen-activated protein kinases (MAPK) signaling pathway.^9^

Here, we demonstrate NF*κ*B signaling pathway involvement in the pathophysiology of the *dy*^*2J*^*/dy*^*2J*^ mouse model, mediating decreased apoptosis and promoting muscle cell survival following Losartan treatment. Reduced apoptosis and pro-survival NF*κ*B target genes activation following treatment suggest a key regulatory role for the NF*κ*B signaling pathway in this disorder.

## Results

### Losartan treatment modifies TNF-*α* expression

**Figure 1:**
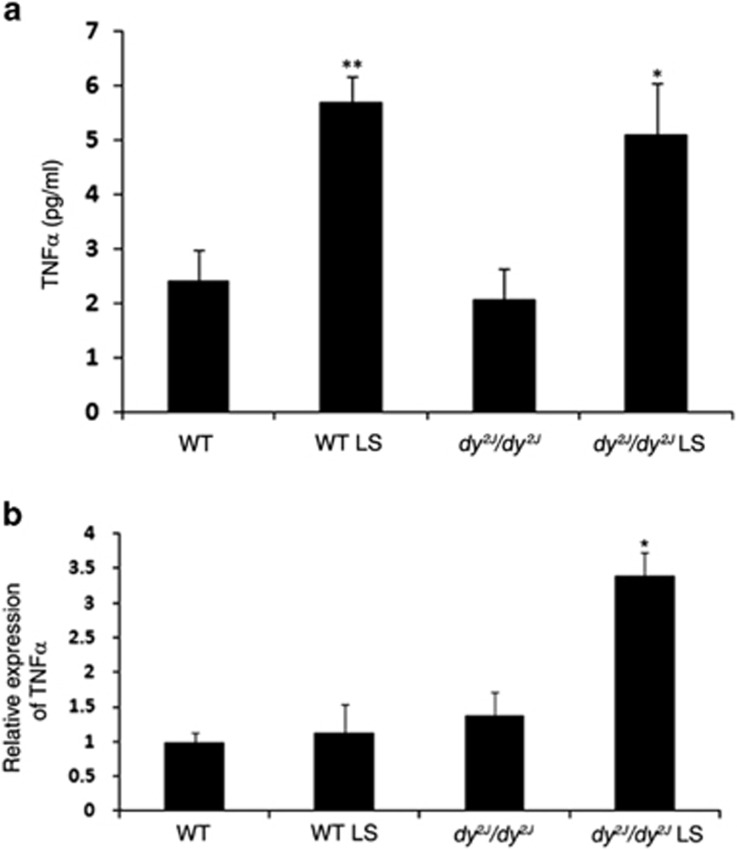
TNF-*α* activity in *dy*^*2J*^*/dy*^*2J*^ and WT mice following Losartan treatment. (**a**) Losartan significantly increased serum TNF-*α* level in treated compared with untreated *dy*^*2J*^*/dy*^*2J*^ mice (*P<0.05). Losartan also increased TNF-*α* levels in treated compared with untreated WT mice (**P<0.0005). Each bar represents the mean±S.E.M. of nine mice at 19 weeks of age. (**b**) Total RNA was extracted from Hind limb muscles of WT and *dy*^*2J*^*/dy*^*2J*^ mice. Quantitative real-time PCR (TaqMan) of *TNF-α* mRNA expression levels was determined. Significant increased mRNA level of *TNF-α* was noted upon Losartan treatment in *dy*^*2J*^*/dy*^*2J*^ mice. Expression levels were normalized to the housekeeping gene, TATA box binding protein (*TBP*) mRNA level (*P<0.05). Results represent the mean±S.E.M. of five mice

The tumor necrosis factor alpha (TNF-*α*) serum level was significantly increased in treated *dy*^*2J*^*/dy*^*2J*^ mice compared with untreated *dy*^*2J*^*/dy*^*2J*^ mice (5.1±0.96 pg/ml *versus* 2.05±0.58 pg/ml; *P<0.05). TNF-*α* serum level was also significantly increased in Losartan-treated WT mice (treated: 5.69±0.49 pg/ml *versus* untreated: 2.42±0.56 pg/ml; **P<0.0005). These results are presented in Figure 1a. As these results were unexpected, we further examined the effect of Losartan on TNF-*α* transcript levels, using quantitative real-time PCR (TaqMan). The *TNF-α* mRNA level was unchanged in untreated *dy*^*2J*^*/dy*^*2J*^ mice compared with WT groups, but was significantly increased in treated *dy*^*2J*^*/dy*^*2J*^ mice hind limb muscles (*P<0.05; Figure 1b). Since TNF-*α* induces NF*κ*B target gene expression, we analyzed NF*κ*B activity and its downstream effects following Losartan treatment in more detail.

### Losartan treatment altered NF*κ*B activation

**Figure 2:**
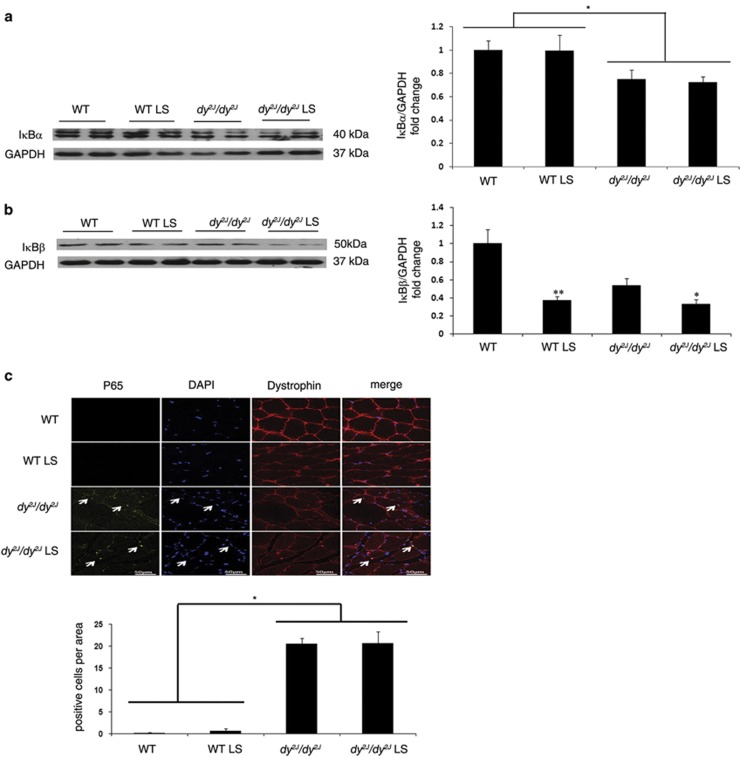
NF*κ*B signaling pathway activity in *dy*^*2J*^*/dy*^*2J*^ and WT mice. (**a**) Representative western blot gel and densitometry graph of NF-kappa-B inhibitor alpha (IkB-*α*) expression in WT and *dy*^*2J*^*/dy*^*2J*^ mice. Significant reduction in IkB-*α* was noted in untreated and treated *dy*^*2J*^*/dy*^*2J*^ mice compared with WT groups (*P<0.005). (**b**) Representative western blot gel and densitometry graph of NF-kappa-B inhibitor beta (IkB-*β*) expression in WT and *dy*^*2J*^*/dy*^*2J*^ mice. Significant reduction in IkB-*β* was noted in treated compared with untreated *dy*^*2J*^*/dy*^*2J*^ mice (*P<0.05). Losartan treatment was also associated with decreased IkB-*β* in treated WT mice compared with the untreated group (**P<0.005). Results of IkB-*α* and IkB-*β* levels were obtained from densitometric analysis and expressed as ratio of IkB-*α*-*β*/GAPDH and as change fold over control (WT group). These results represent three independent experiments. Each bar represents the mean±S.E.M. of 12 mice for IkB-*α* and 11 mice for IkB-*β*. (**c**) Intracellular localization of p65 using Immunofluorescence analysis. Expression of p65 was analyzed using anti-Alexa-647 (yellow fluorescence) antibody. Dystrophin staining as a skeletal muscle marker was analyzed using anti-cy2 (red fluorescence) antibody, and DAPI staining (blue fluorescence) was used as nuclear staining. When p65 protein is localized to the muscle nucleus, Alexa-647, cy2, and DAPI are merged. The quadriceps muscle of untreated and treated *dy*^*2J*^*/dy*^*2J*^ mice showed nuclear localization of p65 demonstrating NF*k*B activation. Lack of p65 in the nucleus was illustrated in untreated and treated WT groups. Scale bar, 50* μ*m. Each bar represents the mean±S.E.M. of five fields per mice of six mice in the WT groups and seven mice in the *dy*^*2J*^*/dy*^*2J*^ groups (*P<0.00001)

Using western blot analysis we found significantly decreased expression of the classic NF*κ*B inhibitor, I*κ*B-*α* protein, in hind limb muscles of both treated and untreated *dy*^*2J*^*/dy*^*2J*^ mice compared with WT mice (untreated *dy*^*2J*^*/dy*^*2J*^: 0.74±0.07-fold *versus* untreated WT: 1±0.08-fold; *P*<0.005; and treated *dy*^*2J*^*/dy*^*2J*^: 0.72±0.05-fold *versus* treated WT: 0.99±0.13-fold; *P*<0.005). However IκB-*α* protein expression was unchanged in *dy*^*2J*^*/dy*^*2J*^ mice following Losartan treatment, indicating pre-existing NF*κ*B activation in untreated *dy*^*2J*^*/dy*^*2J*^ mice (Figure 2a).

We next measured protein expression of IκB-*β*, an additional classic NF*κ*B inhibitor. Only following Losartan treatment was IκB-*β* expression significantly decreased in both *dy*^*2J*^*/dy*^*2J*^ and WT mice (treated *dy*^*2J*^*/dy*^*2J*^:0.32±0.05-fold *versus* untreated *dy*^*2J*^*/dy*^*2J*^: 0.53±0.07-fold; *P<0.05; treated WT: 0.37±0.04-fold *versus* untreated WT: 1±0.15-fold; **P<0.005) (Figure 2b). Thus in untreated *dy*^*2J*^*/dy*^*2*^ NF*κ*B activation follows decreased expression of IκB-*α*, however after Losartan treatment NF*κ*B activation follows decreased expression of both inhibitors; IκB-*α* and IκB-*β.*

In the next step, immunofluorescence staining of quadriceps muscles revealed significantly higher accumulation and co-localization of NFkB p65 subunit in the muscle nucleus of treated and untreated *dy*^*2J*^*/dy*^*2J*^ mice, compared with WT mice (untreated *dy*^*2J*^*/dy*^*2J*^: 20.57±1.25% *versus* WT: 0.166±0.166% *P*<0.00001 and treated *dy*^*2J*^*/dy*^*2J*^: 20.71±2.59% *versus* WT: 0.66±0.49% *P*<0.00001) (Figure 2c). These findings confirm NF*κ*B activation in *dy*^*2J*^*/dy*^*2J*^ mice with and without treatment.

### Losartan treatment upregulates several pro-survival NF*κ*B target genes

**Figure 3:**
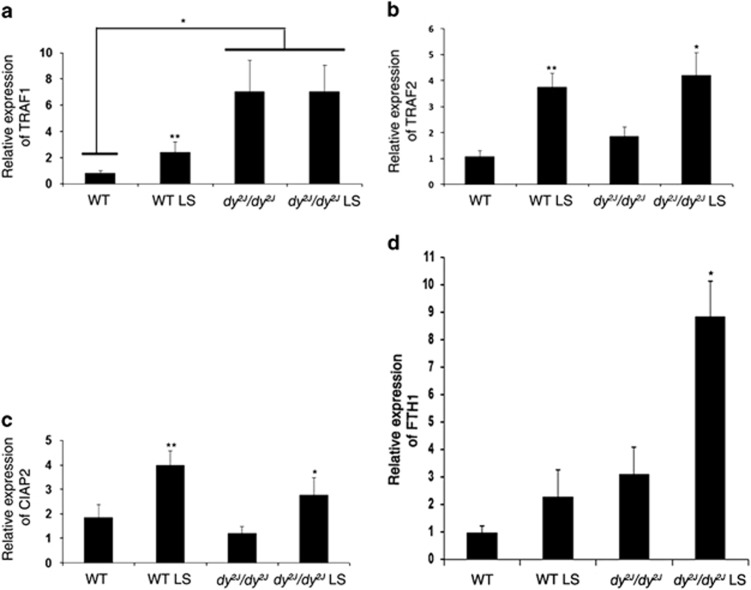
Losartan modified pro-survival/anti-apoptotic NF*κ*B signaling target genes. Total RNA was extracted from Hind limb muscles of WT and *dy*^*2J*^*/dy*^*2J*^ mice. Quantitative real-time PCR (TaqMan) of (**a**) *TRAF1*, (**b**) *TRAF2*, (**c**) *cIAP2*, and (**d**) *FTH1* mRNA expression levels were determined. A significantly increased mRNA level of the anti-apoptotic gene *TRAF1* was noted in treated WT and in both treated and untreated *dy*^*2J*^*/dy*^*2J*^ compared with untreated WT mice (*P<0.01,** *P*<0.05). Significantly increased mRNA levels of the anti-apoptotic genes, *TRAF2* and *cIAP2*, were noted in Losartan treated WT and *dy*^*2J*^*/dy*^*2J*^ mice (*TRAF2*; *P<0.005, **P<0.05 and *cIAP2*; *P<0.01, **P<0.05). Significantly increased mRNA levels of the anti-apoptotic genes *FTH1* were noted in Losartan-treated *dy*^*2J*^*/dy*^*2J*^ mice (*P<0.01). Expression levels were normalized to the housekeeping gene, TATA box binding protein (*TBP*) mRNA level. Results represent the mean±S.E.M. of five mice for *TRAF1*, *TRAF2*, *cIAP2*, and *FTH1*

Since NF*k*B appears to be involved in the regulation of both apoptosis and cell survival, we examined the effect of Losartan on NF*k*B target genes using quantitative real-time PCR (TaqMan). Anti-apoptotic NF*k*B target genes TNF receptor-associated factor 1 and TNF receptor-associated factor 2 (*TRAF1* and *TRAF2*), cellular inhibitor of apoptosis (*cIAP2*), and Ferritin heavy chain (*FTH1*) were analyzed. We found that mRNA expression of *TRAF1*, an adaptor protein required for optimal anti-apoptotic NF*κ*B activation, was significantly increased in treated WT and in both treated and untreated *dy*^*2J*^*/dy*^*2J*^ compared with the untreated WT mice (*P<0.01,**P<0.05; Figure 3a). Because *TRAF1* recruits *TRAF2* and cIAPs to activate the anti-apoptotic process, we next measured the transcript levels of *TRAF2* and *cIAP2* genes. *TRAF2* and *cIAP2* genes were significantly increased in hind limb muscles of both *dy*^*2J*^*/dy*^*2J*^ and WT mice following treatment (*TRAF2*; *P<0.005, **P<0.05 and *cIAP2*; *P<0.01, **P<0.05) (Figures 3b and c). *FTH1* gene expression was significantly increased following treatment in *dy*^*2J*^*/dy*^*2J*^ mice (*P<0.01), with no significant increase in the WT mice (Figure 3d). All of these findings suggest that Losartan upregulates NF*κ*B pro-survival target genes.

### Losartan treatment increases anti-apoptotic protein BCL-2 expression and decreases the pro-apoptotic protein Caspase-3 expression

**Figure 4:**
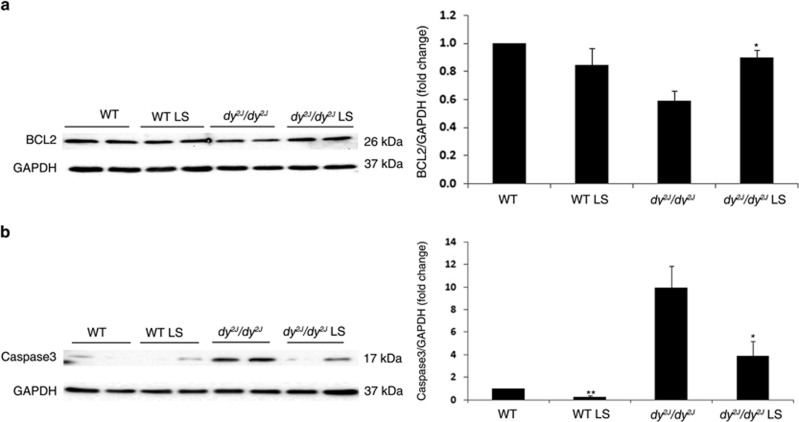
Increased protein expression of anti-apoptotic BCL-2 and decreased protein expression of pro-apoptotic Caspase 3 following Losartan treatment. (**a**) Representative western blot gel and densitometry graph of BCL-2 expression in WT and *dy*^*2J*^*/dy*^*2J*^ mice. A significantly higher BCL-2 protein expression level was noted in treated compared with untreated *dy*^*2J*^*/dy*^*2J*^ mice (*P<0.01). (**b**) Representative western blot gel and densitometry graph of Caspase-3 expression in WT and *dy*^*2J*^*/dy*^*2J*^ mice. A significantly lower Caspase-3 protein expression level was noted in treated compared with untreated *dy*^*2J*^*/dy*^*2J*^ mice (*P<0.0001). Results of BCL-2 and Caspase-3 levels were obtained from densitometric analysis and expressed as ratio of BCL-2 and Caspase-3 to GAPDH and as change fold over control (WT group). These results represent three independent experiments. Each bar represents the mean±S.E.M. of 12 mice for BCL-2 (*P<0.01) and 12 mice for Caspase-3 (*/**P<0.0001)

Next using western blot analysis we examined the hind limb expression of B-cell lymphoma 2 (BCL-2), an anti-apoptotic protein. BCL-2 expression was significantly higher in Losartan treated compared with untreated *dy*^*2J*^*/dy*^*2J*^ mice (treated *dy*^*2J*^*/dy*^*2J*^: 0.90±0.052-fold *versus* untreated *dy*^*2J*^*/dy*^*2J*^: 0.59±0.071-fold; *P<0.01). There was no significant difference in BCL-2 expression between treated and untreated WT mice (Figure 4a).

As for the protein expression level of the pro-apoptotic protein Caspase-3, Losartan treatment reduced significantly its expression in treated compared with untreated *dy*^*2J*^*/dy*^*2J*^ mice (treated *dy*^*2J*^*/dy*^*2J*^: 3.9±1.3-fold *versus* untreated *dy*^*2J*^*/dy*^*2J*^: 10±1.9-fold; *P<0.0001) and in treated compared with untreated WT mice (treated WT: 0.3±0.1-fold *versus* untreated WT: 1±0.02-fold; **P<0.0001 Figure 4b). Taken together, these results suggest that Losartan treatment modifies NF*κ*B signaling toward pro-survival/anti-apoptotic pathway.

### Losartan reduces TUNEL-positive muscle cells

**Figure 5:**
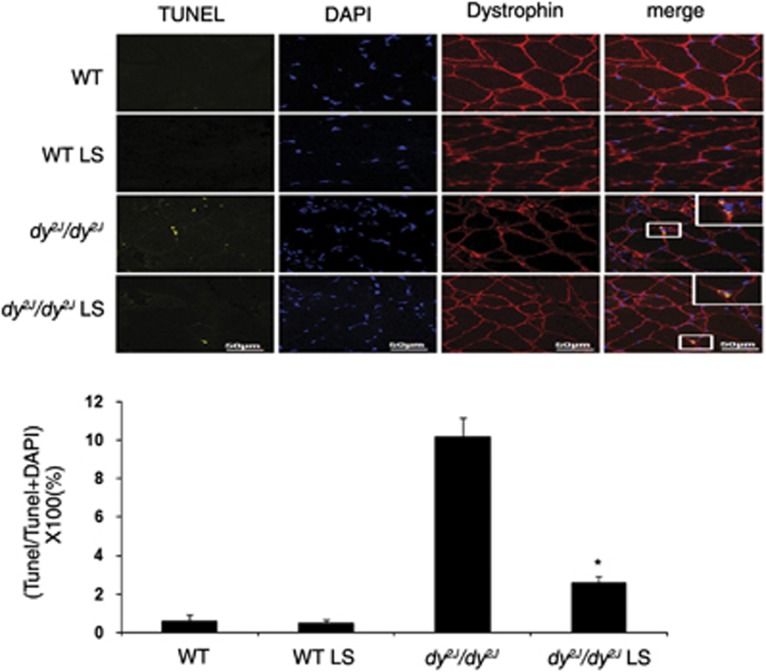
Losartan decreased apoptosis in *dy*^*2J*^*/dy*^*2J*^ mice muscle. Expression of apoptosis was analyzed using TUNEL assay with an *In Situ* Cell Death Detection Kit. TUNEL-positive cells were stained in yellow. Dystrophin, as muscle marker, was analyzed using anti-Alexa-647 antibody (red), and DAPI staining (blue) was used as nuclear staining. The quadriceps muscle of Losartan-treated *dy*^*2J*^*/dy*^*2J*^ mice showed significant reduction in TUNEL-positive cells compare with the untreated mice. Almost no TUNEL-positive cells were illustrated in untreated and treated WT groups. Scale bar, 50* μ*m. Each bar represents the mean±S.E.M. of five fields per mice of six mice in the WT groups and seven mice in the *dy*^*2J*^*/dy*^*2J*^ groups (*P<0.0001)

In order to confirm NF*κ*B involvement in apoptosis signaling, we used *in situ* DNA nick-end labeling (TUNEL), DNA fragmentation assay TUNEL analysis (Figure 5) showed significant reduction of TUNEL-positive cells in quadriceps muscles of Losartan treated compared with untreated *dy*^*2J*^*/dy*^*2J*^ mice, indicating apoptosis (treated *dy*^*2J*^*/dy*^*2J*^: 2.6±0.35% *versus* untreated *dy*^*2J*^*/dy*^*2J*^: 10.14±1% *P<0.0001). Almost no TUNEL-positive cells were found in untreated and treated WT groups.

## Discussion

CMDs are genetically heterogeneous diseases, which result in severe disability and premature death. Muscle fibrotic tissue accumulation and progressive skeletal muscle strength reduction characterize both children and the *dy*^*2J*^*/dy*^*2J*^ mouse model of MDC1 A, one of the most frequent forms of CMD. We previously showed that Losartan treatment significantly increased both fore and hind limb muscle strength, with reduced collagen accumulation and fibrotic markers, in *dy*^*2J*^*/dy*^*2J*^ mice skeletal muscle. This clinical and histological improvement was associated with TGF-*β* and MAPK signaling pathway inhibition, as Losartan was associated with reduced expression of the regulatory Smad; P-Smad2 and 3 and increased expression of the inhibitory Smad; Smad7. Furthermore, Losartan was associated with significant reduction of the three parallel MAPK signaling pathways P-ERK1/2, P-JNK, and P-p38.^9^

Our current study sheds new light on the NF*κ*B signaling pathway and its involvement in the pathophysiology of the *dy*^*2J*^*/dy*^*2J*^ mouse model of MDC1A, mainly through a new insight into the apoptosis pathway. In addition, these data reveal a new role for Losartan with regard to NF*κ*B signaling and apoptosis in this disorder.

In this study, while evaluating Losartan's effect on cytokine levels in mice serum, we found an unexpected significant increase in the TNF-*α* level following treatment in both WT and *dy*^*2J*^*/dy*^*2J*^ mice (Figure 1a). TNF-*α* is known to mediate a variety of cellular responses including inflammation, necrosis, fibrosis, and apoptosis.^27^ One major role of TNF-*α* is stimulation of the NF*κ*B signaling pathway.^28, 29^ We therefore further investigated the NF*κ*B signaling pathway involvement in MDC1A pathology.

NF*κ*B is a transcription factor that in its resting state binds to inhibitory I*κ*B proteins, keeping it inactive and localized to the cytoplasm. Following stimulation NF*κ*B detaches from its inhibitors. The resulting free NF*κ*B translocates into the nucleus where it activates or represses target genes.^17, 30^ Here we demonstrated NF*κ*B activation in both treated and untreated *dy*^*2J*^*/dy*^*2J*^ mice through IκB-*α* protein reduction and p65 (NF*k*B subunit) transcription factor accumulation in the nucleus of skeletal muscle cells (Figures 2a and c). Losartan-treated mice showed NF*k*B activity via reduction of an additional inhibitor, I*κ*B-*β* (Figure 2b). These results may indicate different branches in the NF*k*B pathway are activated upon Losartan treatment compared with untreated *dy*^*2J*^*/dy*^*2J*^ mice.

Previous studies have addressed Losartan's role in inhibition of NF*κ*B inflammatory processes. They showed Losartan reduces and inhibits NF*κ*B activity in muscle cells from porcine coronary artery,^31^ and suppresses inflammation in aged rat kidney.^32^ However, NF*κ*B signaling regulates transcriptional programs that are essential for the development and maintenance of the skeletal system,^33^ epithelium,^34^ and immune system,^35^ which in turn impacts differentiation, proliferation, cell death, and survival.^36, 37^ Therefore, NF*κ*B signaling pathways have a multifaceted regulatory role that can either mediate apoptosis or anti-apoptotic routes. NF*κ*B signaling can also engage the Caspase signaling pathway to mediate cell apoptosis.^38, 39^ On the other hand, it can activate TNF receptor-associated factors (TRAFs) and cellular inhibitor of apoptosis (CIAPs) to suppress cell death.^21, 24, 25, 40^

In Duchenne muscular dystrophy, NF*κ*B activation is perceived as contributing to the deterioration of skeletal muscle pathology and skeletal muscle loss.^18^ For example, deletion of a single allele of NF*k*B (RelA/p65 subunit) was sufficient to considerably reduce infiltration of macrophages, fiber necrosis and calcification in dystrophic muscle in mdx mice (DMD mouse model). In addition, NF*κ*B inhibition augmented the regeneration of mdx mice myofibers.^41, 42^ Furthermore, overexpression in skeletal muscle of A20 protein, a potent negative regulator of NF*κ*B, reduced chronic inflammation and muscle degeneration in mdx mice.^43, 44^

However, in the current study, NF*κ*B activity following Losartan treatment maintained survival and anti-apoptotic effects in *dy*^*2J*^*/dy*^*2J*^ mice. Losartan treatment was associated with increased mRNA expression of pro-survival genes *TRAF1*, *TRAF2*, *CIAP2*, and *FTH-1* (Figures 3a and d). These findings support other studies findings showing gene expression of TRAFs and CIAPs following NF*κ*B activation, lead to inhibition of TNF-*α* induced cell death.^29, 36, 45, 46^ Cells lacking c-IAPs through genetic ablation or treated with IAPs antagonists have been shown to be more sensitive to TNF-*α* induced cell death through decreased NF*κ*B survival mechanism.^25^ Losartan's role in apoptosis inhibition was further demonstrated by a significant increase of anti-apoptotic BCL-2 protein expression in skeletal muscle of treated *dy*^*2J*^*/dy*^*2J*^ mice (Figure 4a). BCL-2 has been shown to have an important role in increasing lifespan, growth rate, and reducing apoptosis following muscle-specific overexpression in *Lama2* null mice.^47, 48^ Furthermore, overexpression of BCL-2 was found to ameliorate muscle weakness and reduce apoptosis in oculopharyngeal muscular dystrophy (OPMD) mouse model.^49^ As for DMD, transgenic overexpression of BCL-2 did not improve muscle pathology in *mdx* mice,^48, 50^ and indeed the effect of Losartan in *mdx* mice is less pronounced than in *dy*^*2J*^*/dy*^*2J*^ mice.^15^

We further investigated the anti-apoptotic effect of Losartan. Losartan treatment significantly decreased the pro-apoptotic protein Caspase-3 in *dy*^*2J*^*/dy*^*2J*^ and WT mice (Figure 4b), and overall apoptosis was significantly decreased in Losartan-treated *dy*^*2J*^*/dy*^*2J*^ mice as demonstrated by TUNEL-positive skeletal muscle cell reduction (Figure 5).

All of the above data together propose an important role for NF*κ*B signaling in the pathophysiology of MDC1A, mainly in terms of its contribution to the apoptosis process. On the basis of current findings, we suggest that Losartan treatment shifts NF*κ*B signaling to favor the survival route *versus* inflammation, fibrosis and apoptosis/damage in the *dy*^*2J*^*/dy*^*2J*^ mouse model of MDC1A.

**Figure 6:**
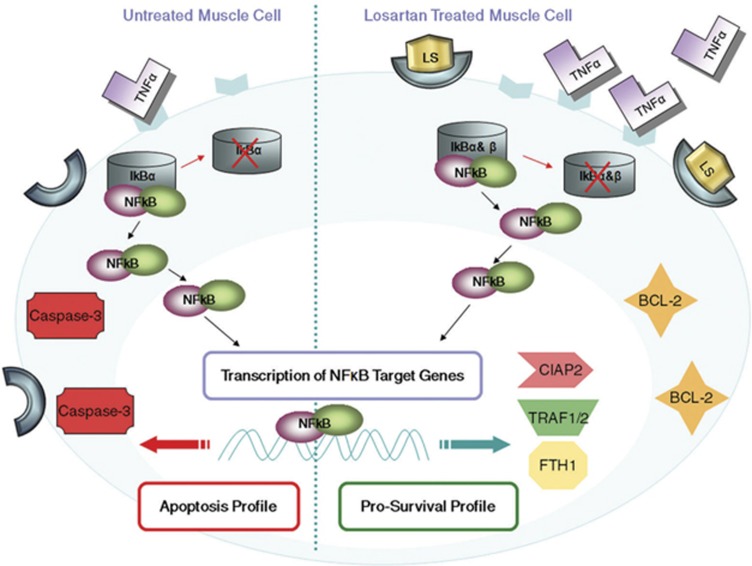
Proposed model for NF*k*B as a key regulator in the survival path of muscle following Losartan treatment. Losartan treatment increases TNF-*α*, which in turn activates NF*κ*B through I*κ*B-*α* and I*κ*B-*β* degradation, p65 nuclear accumulation and upregulation of the pro-survival genes: *TRAF1*, *TRAF2*, *CIAP2*, and *FTH1* in addition to anti-apoptotic BCL-2 protein, to mediate the anti-apoptotic effect of NF*κ*B in skeletal muscle of the *dy*^*2J*^*/dy*^*2J*^ mouse model of MDC1A. In addition, Losartan treatment results in decreased expression of the pro-apoptotic protein Caspase-3

We therefore suggest a model regarding NF*κ*B signaling activity following Losartan treatment. Losartan treatment results in increasing TNF-*α*, which in turn activates NF*κ*B through IκB-*α* and I*κ*B-*β* degradation, p65 nuclear accumulation and upregulation of pro-survival genes and proteins to mediate the anti-apoptotic effect of NF*κ*B in the *dy*^*2J*^*/dy*^*2J*^ mouse model of MDC1A (Figure 6).

In muscular dystrophies there are indications that apoptosis, beside necrosis, may contribute to muscle loss and dysfunction. In human and mouse models of muscular dystrophy signs of muscle cell death by apoptosis have been documented but not discussed in detail.^16, 21, 51, 52^ In this study, we show for the first time that apoptosis has an important role in *dy*^*2J*^*/dy*^*2J*^ muscle pathology. Therefore, it seems that therapies designed to include apoptosis inhibition might be beneficial for patients with congenital muscular dystrophy. These new findings support our previous data that demonstrated significant improvement in animal muscle strength and reduced fibrosis following 12 weeks of Losartan treatment. A future more prolonged Losartan study will provide additional information regarding long-term benefit and survival in *dy*^*2J*^*/dy*^*2J*^ mice. This trial provides further support for a Losartan therapeutic trial in children with MDC1A.

## Materials and Methods

### Mice population and treatment

Muscle tissues for this study were obtained from the mice used in a previous Losartan study^9^ as follows; C57BL/6J Lama2dy-2J heterozygote mice (Jackson Laboratories, Bar Harbor, ME, USA) were bred at the Hebrew University specific pathogen-free animal housing facility. The joint ethics committee of Hebrew University and Hadassah Medical Center (accredited by AAALAC) approved the study protocol for animal welfare (permit number: 122.03-04). Mice were maintained under standard conditions, 23±1 °C, 12- h light cycle (0700–1900 h), with *ad libitum* access to food and drink. Delineation between the Lama2dy-2 J (*dy*^*2J*^*/dy*^*2J*^) affected mice, heterozygous for the LAMA2 gene mutation, and wild-type C57BL/6 J (WT) mice was detected by PCR.^53^ WT and *dy*^*2J*^*/dy*^*2J*^ mice received 0.6 g/l Losartan (Merck Sharp & Dohme, West Point, PA, USA) in their drinking water or placebo as a control, The mice were treated for 12 weeks from 6 weeks of age (*n*=12/group; each group consisted of 6 male and 6 female mice).

### Cytokines

A commercial BDTM Cytometric Bead Array Mouse Th1/Th2/Th17 Cytokine Kit (CBA) (lot: 77184, BD Biosciences, San Jose, CA, USA) was used to determine the levels (pg/ml) of TNF-*α* in serum samples, according to the manufacturer's instructions. Fluorescence was analyzed using a flow cytometer (BDTM LSR II flow cytometer system; BD Biosciences) and the cytokine level was determined using a BD CBA Software (BD Biosciences).

### Western blot analysis

Western blot analysis was performed as previously described.^8^ Immunoblotting was performed using the following antibodies: anti I*κ*B-*α* and I*κ*B-*β* (Santa Cruz Biotechnology, Santa Cruz, CA, USA), anti-BCL-2 (Santa Cruz Biotechnology) and anti-Caspase-3 (Sigma-Aldrich, St Louis, MO, USA). The antibodies were used according to standard procedures. Equal protein loading of blots was confirmed by immunoblotting of glyceraldehyde-3-phosphate dehydrogenase (GAPDH; Santa Cruz Biotechnology). Densitometry of the bands was obtained by chemi DOC XRS+ image lab software (Bio-Rad Laboratories, Hercules, CA, USA). Results are presented as fold of change over control (untreated WT group), which is defined as 1.

### Real-time quantitative PCR

RNA extraction was performed with Tri Reagent (Sigma-Aldrich) according to the manufacturer's instruction. cDNA was reverse transcribed from total RNA with random primers using the High-Capacity cDNA Reverse Transcription Kit with RNase inhibitor according to the manufacturer's instruction (Applied Biosystems, Foster City, CA, USA). mRNA expression was quantified by TaqMan (Applied Biosystems) using the Illumina high-performance Eco Real-Time PCR system. Quantitative real-time PCR (TaqMan) assays were conducted in triplicates, and standard deviation was used to calculate error bars.

### Immunofluorescence

Quadriceps muscles were isolated and fixed in acetone for 1 h. The quadriceps muscle tissues were sliced into 8 *μ*m cross sections after being embedded in OCT. The mounted sections were washed three times in phosphate-buffered saline (PBS), blocked with 1% bovine serum albumin and probed with anti-p65 rabbit antibody (Abcam, Cambridge, UK) and with anti-dystrophin mouse antibody (Santa Cruz Biotechnology). After three washes in PBS, the sections were incubated with Alexa fluor 647-conjugated affini-pure donkey anti-rabbit IgG (H+L) and with Cy2-conjugated affini-pure donkey anti-mouse IgG (H+L) (Jackson ImmunoResearch, West Grove, PA, USA). After three more washes in PBS, coverslips were mounted on glass slides with a DAPI-containing mounting medium (Vector Laboratories, Burlingame, CA, USA). Fluorescence analysis was performed using a Zeiss LSM 710 confocal laser scanning system (Carl Zeiss MicroImaging GmbH, Jena, Germany). The P65-positive fluorescent cells were counted under a fluorescent microscope, and the numbers were expressed as the percentage of total P65 area cells±S.D. A negative control without anti-p65 rabbit antibody (Abcam) was also performed.

### Tunel staining

Quadriceps muscles sections were assessed by terminal deoxynucleotidyltransferase-mediated dUTP nick end labeling (TUNEL) assay with an *In Situ* Cell Death Detection Kit (Roche Diagnostic, Indianapolis, IN, USA). Muscles were isolated and fixed in acetone for 1 h; the tissues were sliced into 8 *μ*m cross sections after being embedded in OCT. Afterwards washed twice in PBS. Each slice was embedded with 100 *μ*l permeabilisation solution, 0.1% Triton X-100 for 2 min, washed twice in PBS and the tissue sections were labeled and stained with the TUNEL reaction mixture (label plus enzyme solutions) for 60 min at room temperature and washed twice with PBS. Then the slices were probed with anti-dystrophin mouse antibody (Santa Cruz Biotechnology), and after three more washes in PBS the sections were incubated with Alexa fluor 647-conjugated affini-pure donkey anti-mouse IgG (H+L) (Jackson ImmunoResearch). After three more washes in PBS, coverslips were mounted on glass slides with a DAPI-containing mounting medium (Vector Laboratories). The apoptotic fluorescent cells were counted under a fluorescent microscope, and the numbers were expressed as the percentage of total Tunel area cells±S.D. A negative control without enzyme treatment and a positive control with DNase I (Roche Diagnostic) treatment were also performed.

### Statistical analysis

All data are expressed as mean and standard error of the mean. Statistical analysis for direct comparison between two groups was performed by unpaired Student's *t* test. Significance was set at *P*<0.05 for all comparisons.

## Abbreviations

MDC1A: congenital muscular dystrophy type 1A
CMD: congenital muscular dystrophy
DMD: Duchenne muscular dystrophy
OPMD: oculopharyngeal muscular dystrophy
LGMDs: Limb girdle muscular dystrophies
NF*κ*B: Nuclear factor kappa B
TGF-*β*: transforming growth factor beta
MAPK: Mitogen- activated protein kinases
TRAF1: TNF receptor-associated factor 1
TRAF2: TNF receptor-associated factor 2
cIAP2: cellular inhibitor of apoptosis
FTH1: Ferritin heavy chain
TBP: TATA box binding protein
TNF-*α*: tumor necrosis factor alpha
I*k*B-*α*: NF-kappa-B inhibitor alpha
I*k*B-*β*: NF-kappa-B inhibitor beta
BCL-2: B-cell lymphoma 2
